# Legal Notes for Institutional Workers

**Published:** 1915-02-13

**Authors:** 


### LEGAL NOTES FOR INSTITUTIONAL WORKERS.

### Relation of Doctors to Insurance Committees.

We referred recently to a decision on a question between a medical man and the National Health Insurance Commissioners. Now the case of *O'Driscoll* v. *Manchester Insurance Committee*, 31 T. L. R. 103, settles another point as to the relationship of doctors to the Committees under that statute. The immediate and practical result of that particular case is that a doctor's fees in the hands of an Insurance Committee may be attached by his creditors. But two general principles were established, which fall to be noted here. The first relates to the argument put forward by the Insurance Committee itself, that it was contrary to public policy that such moneys should be attachable. This was based on analogy with the familiar principle that due to considerations of public policy the salaries of officers in the service of the Crown cannot be attached. But the Court at once definitely ruled that that doctrine had no application to the case of a National Health Insurance panel doctor. The next point was whether there was in fact any money due by the Committee to the doctor that could be attached, seeing that the fund out of which the defendant Committee had to pay for medical services was not a fund of their own, but was collected and distributed by the Insurance Commissioners, the only duty of the Committee being to agree with doctors to render medical services. The Court, however, held that when the Committee had received money from the Commissioners, a debt was due and accruing from the Committee to every doctor who had done work for them. Before the Committee received the money there was no such debt, but as soon as the money came in there was one. Attachment of such a debt by a creditor of a doctor was therefore competent and effectual.

### Miner's Nystagmus.

One of the industrial diseases scheduled to the Workmen's Compensation Act is miner's nystagmus, and it has been the subject of two cases recently which we think it useful, and indeed necessary, that certifying surgeons under that statute should note. In *McGowan* v. *Merry and Cunninghame, Ltd.* (1914), S. L. T. 298, it was found on the evidence submitted that the disablement of the miner from nystagmus was due in part to the nature of his employment before the date which the certifying surgeon certified as the date on which disablement commenced, and partly to the nature of his employment after that date. The Court held that this was sufficient to entitle the workman to compensation, and that in view of the cases already decided it was hopeless to maintain that the workman would have no remedy unless the disease in question was the sole cause of his disablement. It is enough for him to establish that the disease was one of the causes of his disablement.

In the other case, *McTaggart* v. *Barr and Son* (1914), 2 S. L. T. 430, it is necessary to understanding of the point decided to refer to the relevant sections of the statute. It provides (Section 8 (4)) that in the case of industrial diseases the date of disablement shall be such date as the certifying surgeon certifies as the date on which the disablement commenced, or if he is unable to certify such a date, the date on which the certificate is given; and then by Section 8 (2) it is enacted that if the workman at or immediately before the date of disablement was employed in an industry in which he contracted an "industrial disease," such disease, unless where the certifying surgeon certifies that in his opinion the disease was not due to the nature of the employment, shall be deemed to be due to the nature of that employment, unless the employer proves the contrary.

Now it must be recollected that in the case mentioned the workman was disabled by miner's nystagmus on March 26, 1914. He had been engaged in mining from May 15, 1913, to July 11, 1913, but had not been working from July 11, 1913, to March 26, 1914. In that state of matters the Court held that the miner had not "immediately before the date of disablement" been employed in mining, and therefore, instead of the onus being thrown on the employer of proving that the disablement was due to mining, it was for the workman to discharge the onus of proving that the disease was due to his employment. An ingenious argument was presented to the Court to the effect that the statutory expression "immediately before," etc., referred to sequence of employment, and not to sequence in time, and that if there had been no intermediate employment engaged in by the miner between the date of his last employment in mining and the date of disablement, then the last employment in mining, however far distant within the twelve months, must be considered in the sense of the statute to be "immediately before" the date of disablement. But the Court decided as above.

